# Transitioning from Traditional Academic Decision Making to Patient-Centric Healthcare Choices: The Example of Thyroid Thermal Ablation Techniques for Papillary Thyroid Microcarcinomas

**DOI:** 10.3390/curroncol30110701

**Published:** 2023-10-31

**Authors:** Hervé Monpeyssen

**Affiliations:** Institut de la Thyroïde, 75001 Paris, France; hm.thyroide@gmail.com

Percutaneous thermal ablation techniques (TATs) have contributed to improving thyroid tumor management for almost twenty years. They give the opportunity to treat the thyroid nodule “in situ” while preserving the surrounding healthy thyroid tissue and the surrounding vital structures of the patient’s neck. They were initially dedicated to ablating benign thyroid tumors; yet, it has to be said that they also present a simple, safe and reliable alternative to the management of some selected differentiated thyroid cancers. They contribute to the current therapeutic de-escalation trend and can reasonably be proposed to the thyroid nodule patient, who is nowadays clearly an integral part of the therapeutic decision-making process.

Participating in the evolution of medical practices is undoubtedly one of the most exciting aspects of our medical art. As a matter of fact, looking back over the past fifty years, the thyroid nodule management equation was relatively simple. The presence of a palpable neck lump invariably led to a serum TSH assessment and thyroid scintigraphy, with a non-avid thyroid nodule leading to surgery, usually a total thyroidectomy.

In the case of malignant histology, surgical re-intervention and lymph node dissection 100 mCi radio-metabolic treatment with T4 withdrawal were performed.

The consequence was a high curative rate and a lifelong suppressive therapy.

Then, we witnessed the advent of the following events:

(a) In biology, the development of US hormone assays, postoperative cancer follow-up using serum thyroglobulin level, serum calcitonin level in the detection and monitoring of medullary thyroid cancer.
(b) The extraordinary advancement of multiparametric high-frequency ultrasound and the emergence of the TIRADS scoring system (Thyroid Imaging Reporting and Data System).
(c) The high accuracy of fine needle aspiration cytology (FNAC) and the Bethesda System.

The mutual reinforcing effect of these techniques has revolutionized our understanding of thyroid nodules. The standardization of the different TIRADS scores is currently underway, and the third edition of the Bethesda System is now available.

(d) Molecular biology, including the analysis of cytological samples, has taught us about tumor-generating mechanisms and paved the way for targeted therapies.
(e) Thyroid scintigraphy has become quantified, and the use of the Recombinant Human Thyrotropin (rh-TSH) has ruled out the need for withdrawal of levothyroxine, which was often poorly tolerated by the surgical patient.
(f) Tyrosine kinase inhibitors (TKIs) have proven their efficacy in case of resistance to conventional treatments, and immunotherapy shows promising results.
(g) Histopathology assessments have significantly evolved, and a recent update of the thyroid tumor atlas is now available.
(h) Lastly, surgical techniques have become less extensive thanks to ultrasound accuracy, and alternative scar-minimizing approaches including the Robotic or Transoral Endoscopic Thyroidectomy Vestibular Approach (TOETVA).

Simultaneously, prospective cohort studies have shown that treatments have been overly aggressive, and there is no benefit to survival or complications by treating all tumors with a heavy protocol (although it remains obviously applicable in cases of highly aggressive tumors).

Finally, we have come to understand that the patient indeed is at the center of the debate. The patient desires to voice their own opinions, make informed choices, and retain the option to decline a lifelong medication, especially if changes in its formulation may have adverse effects on their health. The patient seeks to be recognized as an active participant in their medical journey. Therefore, a thyroid cancer patient may choose to keep the thyroid gland in place in the absence of evidence of a severe form of cancer. The patient’s satisfaction scale assessment has also become mandatory.

All these elements have contributed to the current trend of therapeutic de-escalation of thyroid cancer. The sacrosanct total thyroidectomy has given way to a more limited surgical lobectomy procedure, and adjuvant radio-iodine therapy is no longer mandatory. Thus, patients are having less operations and irradiation in all cases. The scientific breakthrough of Non-Invasive Follicular Thyroid Neoplasm with Papillary like Nuclear Features (NIFT-P tumors) has reinforced this approach. Also, the experience of the thyroid carcinoma epidemic in Korea has shown the harm of overdiagnosis. Regarding the most common type of thyroid cancers, the Papillary Thyroid Micro Carcinoma (PTMC), two alternatives have thus emerged as a result: Active Surveillance (AS) and Thyroid Thermal Ablation Techniques (TATs).

AS is definitely a reliable solution to be considered in patients with low-risk thyroid carcinoma. The physician should be aware of assessing a work-up of the patient’s condition (age >20 years old, absence of familial thyroid cancer history, lack of previous radiation therapy of the neck, aggressive or unfavorable features, minimal distance to the capsule) and provide biennial tumor monitoring [1].

However, some patients struggle with the idea of being diagnosed with thyroid cancer and not receiving treatment.

Thyroid Thermal Ablation Techniques

The principle of thermal-ablation is the heating of a tissue with the aim of destroying it or limiting its growth or functional characteristics. Above 60 °C, instant cell death can be observed, followed by the creation of a vapor bubble (as far as I know, cryotherapy has never been used for thyroid treatment). Currently, four techniques areavailable as follows: Laser Ablation (LA), Radiofrequency Ablation (RFA), MicroWave Ablation (MWA), and High Intensity Focused Ultrasound (HIFU). The first three techniques use a specific energy generator, a transmission cable, and an ancillary device introduced into the thyroid tissue. Regarding LA, a fiber delivering light energy is inserted into the thyroid through a Chiba needle. Considering RFA, the electrode (which can be either unipolar or bipolar) generates ionic agitation with frictional heat. MWA delivers energy, which is transmitted by an antenna, leading to an extremely rapid friction of water molecules, with a subsequent thermal heating effect and no necessity of pads. High-Intensity Focused Ultrasound (HIFU) is the only technique that does not use an emissary. Ultrasound beams are focused to heat the tissue in a target area, leading to the removal of small volume zones to be treated. Currently, it is not recommended by the medical societies as this is less effective than the other three techniques and much more expensive [2]. In most cases, local anesthesia provides analgesia, and the patient is managed on an out-patient or day-hospital basis. The results are very satisfying, with a volume reduction of 75 to 85% at one year follow-up and an acceptable low regrowth percentage (known to be more frequent using LA). Thyroid nodule regrowth is mostly seen in nodules incompletely treated due to their location or volume [3]. As a matter of fact, a thyroid nodule with a volume >40 mL will indeed require at least two successive percutaneous procedures. The patient satisfaction rates commonly exceed those of surgery, especially because serious reported complications are very rare. They may include Horner syndrome (1/1000 cases), permanent vocal cord damage (1/500 cases), and tracheal injury (few reported cases in the literature) [4]. Noteworthy, the most common complication is thyroid nodule rupture (1–2% for all teams), which is due to the transformation of the treated tissue into a liquid form that leaks beyond the thyroid capsule.

This almost always regresses with medical treatment [5]. The effectiveness and reliability of percutaneous thermal ablation in benign thyroid nodules are well established. In 2021, Stella Bernardi et al. published a very interesting paper focused on this subject [6]. Interestingly, in 2020, Enrico Papini et al. published the European Thyroid Association (ETA) Clinical Practice Guidelines for the Use of Image-Guided Ablation in Benign Thyroid Nodules [7]. The primary indication is a single nodule becoming bothersome or rapidly progressing. Treatment can also be considered in a predominant thyroid nodule located in a multinodular goiter, a solid and cystic nodule remaining after ethanol ablation failure, or an autonomous thyroid nodule. Indeed, the recent novelty is that papillary thyroid cancer thermal ablation is also taken into account as an alternative therapy to thyroid surgery [8]. The thyroid carcinoma volume and precise location on a high frequency ultrasound should be very carefully analyzed by expert operators [9]. The number of foci, the absence of extracapsular spread, the presence of an acute angle between the tracheal wall and the nodule, a minimum of 3 mm thickness margin of normal thyroid tissue between the oesotracheal triangle and thyroid cancer, the absence of suspicious metastatic lymph nodes, and the absence of dysphonia are all crucial elements to be determined before a decision had been made by the pluridisciplinary council.

The Role of Expert Ultrasound and TAT Operator

This is evidence that a multiparametric ultrasound is one of the foundations of this minimally invasive approach for a thyroid nodule. It serves the following purposes: the detection of non-palpable tumors, thyroid nodule characterization on B-mode, US Doppler and shear wave elastography (this is particularly relevant for Papillary Thyroid Carcinoma, PTC), accurate guidance for fine-needle aspiration cytology (FNAC) and core needle biopsy of a suspicious thyroid nodule, in situ thyroglobulin/calcitonin measurement of a nodule or suspicious neck lymph node (follicular/medullary carcinomas), depiction of lymph nodes at initial work up and preoperative staging, and follow-up of treated thyroid cancers.

The operator should assess the technical feasibility for thyroid thermal ablation; ideally, this should be done by the operator involved in the percutaneous procedure.

Considering the oncology point of view, achieving a local curative treatment necessitates a skilled operator who will take into account not only the presence of healthy peripheral rim thyroid tissue but also apply safety margins for malignant nodule TAT. This can be done by using multiparametric ultrasound and contrast enhanced ultrasound monitoring [10].

Obviously, the operator has to analyze the proximity of fragile structures in contact with the thyroid tumor, mainly the nerves (vagus nerve, external branch of the superior laryngeal nerve, recurrent laryngeal nerve, middle sympathetic cervical ganglion, brachial plexus branch, or spinal accessory nerve in the case of metastasis treatment), as well as the vascular or visceral structures (trachea and esophagus).

During the Thyroid Thermal Ablation procedure (TAT), monitoring the local anesthesia needle, the hydro dissection needle, the heat device (fiber, electrode, or antenna), the real-time monitoring of the ablation progress, and heat delivery quantification are of the utmost importance. Evaluating the need for hydro dissection is now of paramount importance [11]. This protective technique consists of injecting NaCl or 5% dextrose (G5), either as a single injection or as an infusion into the soft tissues of the neck, to separate the area to be treated from the neck fragile structures such as trachea or neurovascular bundles. It can also be essential for treating isolated neck lymph node metastasis. In cases of nerve discomfort (vocal cord impairment during the procedure), rescue hydro dissection with chilled G5 is used as an efficient therapeutic cooling emergency measure.

Post procedure surveillance will assess tumor evolution (reduction, disappearance, and regrowth) and will look for lymph node involvement.

As percutaneous TAT has shown high reliability and effectiveness in benign thyroid lesions, its potential was logically extended to the oncology field. It is in this way that large retrospective studies, meta-analyses, and two consensus conferences definitively provided its accurate indications and proper limitations [12]. Therefore, thermal ablation is gaining a prominent place in the decisional algorithm of thyroid carcinoma management, namely in cases of low-risk PTC. Indeed, low-risk PTC tumors, including microcarcinomas staged as pT1aN0M0 and pT1bN0M0 PTC, certain selected cases of local cancer recurrence, and non-iodine-avid metastases could be effective therapy options.

However, opinion is critical of the following cases: pT2, pT3, pT4, and N+ carcinomas, aggressive histological types such as the tall-cell variant or hobnail tumors, papillary cancers with BRAF or TERT mutations, and medullary cancers. The European Thyroid Association and Cardiovascular and Interventional Radiological Society of Europe 2021 Clinical Practice Guideline for the Use of Minimally Invasive Treatments in Malignant Thyroid Lesions have addressed the subject [13].

Concerning the three major techniques (LA, RFA, MWA), the results in terms of volume reduction, decrease in serum thyroglobulin level, percentage of regrowth, and occurrence of complications are very promising [14]. The comparison of those results to thyroid surgery does not rule out TAT at all [15]. In most cases, thyroid cancer is not considered as a “human being killer cancer”. The term “papillary thyroid carcinoma” in the histologist’s report in fact does not mean a “death sentence” for the thyroid cancer patient. Apart from anaplastic cancer, some rare aggressive forms, and some metastatic concerns, there is no urgency to treat thyroid cancer. This allows time for a precise and comprehensive evaluation, including an expert ultrasound assessment, possible repeat fine-needle aspiration associated with molecular analysis, and potentially, additional cross-sectional imaging. These findings will further be discussed by the multidisciplinary council (MDC) to inform the patient about the available choices for appropriate personalized therapy.

The patient should revisit his referring thyroidologist to be informed of the MDC conclusions, the constraints, adverse effects, and benefits of each therapeutic option, as well as the follow-up requirements for each option. Depending on the scenario, (a) in the case of active surveillance, the patient has to sign a commitment for regular US neck monitoring; (b) in the case of thermal ablation, the physician will explain the complete process to the patient, and as TAT is a relatively novel technique, the patient should not rely on social media to understand its intricacies; and (c) in the case of surgery, they should discuss the postoperative phase extensively, including the review of the case by the MDC and the potential need for a second surgery and radioiodine treatment.

A summary should be provided to the patient and a duplicate sent to the primary care physician.

Times have changed and responses need to be innovative. Policy choices with respect to organ function preservation and lesser complications do exist. Empowering the patient is crucial in our actual approach to care [16].

In conclusion, in case of papillary microcarcinoma, the following reasons for considering TAT novelty include (a) satisfactory results in terms of reducing tumor volume and addressing patient concerns; (b) the lack of a scar in the neck, namely for female patients concerned about their appearance after treatment; (c) a low complication rate (optimal for both the patient and practitioner); (d) a short out-patient hospital stay, thus minimizing disruption to the daily professional life; (e) a possible repeat of the procedure if needed, allowing for additional treatments or adjustments if the initial outcome is not optimal; (f) the reduced painkiller usage, indicating a relatively comfortable recovery; and (g) a better cost-effectiveness than surgical alternatives, even in an outpatient setting. In some countries, social insurance may cover the procedure’s cost, making it accessible to a broader patient population (Italy for instance).

These advantages taken together definitely make thermal ablation an attractive option for both patients and practitioners. This fits perfectly well with the current trend of patient-centered care [17].
